# A Systems Biology Approach to Characterize Biomarkers for Blood Stasis Syndrome of Unstable Angina Patients by Integrating MicroRNA and Messenger RNA Expression Profiling

**DOI:** 10.1155/2013/510208

**Published:** 2013-05-14

**Authors:** Jie Wang, Gui Yu

**Affiliations:** Department of Cardiology, Guang'anmen Hospital, China Academy of Chinese Medical Science, Beixiange 5, Xicheng District, Beijing 100053, China

## Abstract

Blood stasis syndrome (BSS) has been considered to be the major type of syndromes in unstable angina (UA) patients. The aim of this study was to find the systems biology-based microRNA (miRNA) and mRNA expression biomarkers for BSS of UA. We identified 1081 mRNAs and 25 miRNAs differentially expressed between BSS of UA patients and healthy controls by microarrays. We used DAVID, miRTrail, and the protein-protein interactions method to explore the related pathways and networks of differentially expressed miRNAs and mRNAs. By combining the results of pathways and networks, we found that the upregulation of miR-146b-5p may induce the downregulation of CALR to attenuate inflammation and the upregulation of miR-199a-5p may induce the downregulation of TP53 to inhibit apoptosis in BSS of UA patients. The expression patterns of miR-146b-5p, miR-199a-5p, CALR, and TP53 were confirmed by qRT-PCR in an independent validation cohort including BBS of UA patients, non-BBS of UA patients, and healthy controls. miR-146b-5p, miR-199a-5p, CALR, and TP53 could be significant biomarkers of BSS of UA patients. The systems biology-based miRNA and mRNA expression biomarkers for the BSS of UA may be helpful for the further stratification of UA patients when deciding on interventions or clinical trials.

## 1. Introduction

Unstable angina (UA) constitutes a clinical syndrome subset of the acute coronary syndrome (ACS) and is associated with an increased risk of cardiac death and subsequent myocardial infarction (MI) [1]. UA is diagnosed by electrocardiographic (ECG) ST-segment depression or prominent T-wave inversion and negative biomarkers of necrosis and in an appropriate clinical setting (chest discomfort or angina equivalent). Its pathophysiological origins relate to disruption or erosion of an atherosclerotic plaque and a subsequent cascade of pathological processes that decrease coronary blood flow [2]. UA remains a severe burden on society and family in both industrialized and developing countries. Although anti-ischemic, antiplatelet, and anticoagulant/antithrombotic therapies and early standard coronary revascularization procedures have been used for fighting against UA, there are some drawbacks of current therapeutics, such as aspirin resistance and adverse effects of statin [3, 4]. New thoughts need to be present for developing efficient diagnosis and optimal therapeutics to combat UA. 

Traditional Chinese medicine (TCM) is rapidly gaining attention in the world as sources for discovering new cardiovascular drugs [5–9]. However, simply copying TCM therapy to the treatment of UA is not feasible. Some systematic reviews have shown that the benefits of standard TCM therapies given to UA patients are not always obvious [10, 11]. We think that the main problem is that these clinical trials did not consider the further stratification of UA patients based on TCM syndrome differentiation. Since a single disease can have several kinds of syndromes, it should be treated with different therapies instead of one single therapy [12–14]. According to TCM theory, blood stasis (“*Xueyu*” in Chinese Mandarin) is one of the key pathogenesis of UA. This has been confirmed by a binary logistic regression analysis of a multicenter prospective research on TCM Syndromes in 815 cases of UA [15]. Blood stasis syndrome (BSS) refers to a condition in which any pathological change is characterized by retarded or impeded blood flow. The local manifestations of BSS include mass formation, ecchymosis or petechiae, and stabbing or pricking pain fixed in location and accompanied by tenderness. The general manifestations of BSS include darkish complexion, skin texture becoming thickened, lack of smooth feeling, dark purple tongue with purple spots, and choppy or irregular pulse. Since BSS is one of the most common syndromes in TCM, lots of research has been done about it [16–21]. The grading system in quantifying BSS diagnosis standards has been built by clinical investigation and multifactorial analysis, and regressive analysis as well as differential analysis in China since 1988 [16, 22]. The grading system can be adapted to all kinds of BSS patients, including UA patients.

Among the classic teachings of TCM, it is stated that “when blockage is opened, pain is relieved; when blockage is not opened, pain persists.” If a UA patient has BSS, the treatment should be promoting blood circulation and removing blood stasis (PBCRBS). The concept of PBCRBS is similar to the modern revascularization method, but it is a kind of macroscopic thinking achieved by drugs rather than microscopic mechanism. The benefit of PBCRBS in UA patients with BSS has been demonstrated by increasing clinical evidences in recent years [23, 24]. It again confirms the importance of considering the further stratification of UA based on TCM syndrome differentiation in clinical trials. More importantly, it gives valuable impetus to the hypothesis that BSS of UA in TCM may have its own specific biomarkers. Exploring these biomarkers may facilitate further risk stratification of UA and provide some new therapeutic targets for treating UA. 

MicroRNAs (miRNAs) are endogenous, nonprotein-coding, single-stranded, small RNAs that are generally regarded as negative regulators of gene expression by inhibiting translation and/or promoting messenger RNA (mRNA) degradation [25]. In various cardiovascular diseases, gain- and loss-of-function studies using *in vitro* and *in vivo* models have revealed pathogenic and protective functions of miRNAs; therefore they emerge as interesting novel candidates for the development of miRNA-based therapeutic strategies in cardiovascular disease [26]. Besides their function, recent studies have been demonstrated that miRNAs can circulate in the blood of cardiovascular-diseased patients in a remarkably stable form [27]. The discovery of circulating miRNAs opens up intriguing possibilities to use the circulating miRNAs' patterns as biomarker for cardiovascular diseases. Therefore, circulating miRNAs' patterns are likely to provide supplementary information for investigating biomedical mechanisms of BSS of UA patients and characterizing its biomarkers. 

The purpose of this study was to investigate circulating miRNAs' patterns of BSS of UA patients by a systems biology approach. MiRNA and mRNA expression profilings of peripheral blood mononuclear cells (PBMCs) of BSS of UA patients were compared to ones of PBMCs of healthy controls to identify the differentially expressed miRNAs and mRNAs by using a gene expression oligonucleotide microarray and a microRNA microarray. Bioinformatics analysis was used to find critically deregulated miRNAs and mRNAs involved in pathogenesis of BSS of UA and potential biomarkers. The expression patterns of miRNA and mRNA biomarkers in BSS of UA patients were independently validated by real-time quantitative polymerase chain reaction (qRT-PCR) in an independent validation cohort including BBS of UA patients, non-BBS of UA patients, and healthy controls. 

## 2. Materials and Methods

### 2.1. Patients and Controls

Patients with UA undergoing clinically indicated coronary angiography were consecutively recruited into the study in Guang, Anmen Hospital, Beijing, China. The diagnosis of coronary artery disease (CAD) was confirmed in all patients by coronary angiography showing at least one vessel disease (>50% narrowing of luminal diameter). UA patients were eligible to participate if they had met the American College of Cardiology/American Heart Association (ACC/AHA) criteria for UA [1]. All UA patients (*n* = 65) had experienced ischemic chest pain within the preceding 48 h, including angina pectoris with an accelerating pattern, or prolonged duration (>20 min), or recurrent episodes at rest or within minimal effort, but with no evidence of enzymatic criteria. Transient ST-T segment depression and/or T-wave inversion were present in all cases. In patients undergoing percutaneous coronary intervention, all blood samples were taken before this procedure.

UA patients were diagnosed as BSS or non-BSS according to the grading system in quantifying BSS diagnosis standards. It had 33 items for diagnosis, including symptoms, signs, and laboratory tests (Table 1). Each item had an assigned point. If the grade of 33 items of the diagnostic scale of BSS in a patient was more than 19, the patient could be diagnosed as BSS. If the grade of 33 items in a patient was less than 19, the patient could be diagnosed as non-BSS. The severity of BSS of patients could be assessed by their grades. Although BSS was the major syndrome type of UA, UA also had other syndromes, such as phlegm, qi, or blood deficiency. This study just included UA patients who had only BSS in BSS group. The diagnosis about BSS of UA patients were made by 3 appointed TCM practitioners. Patients were included in the study only if the 3 practitioners reported consistent results. This ensured that all of the selected patients had typical manifestations of BSS. 

Patients who had received thrombolytic therapy in the previous month were excluded from the study. Patients with stable angina, MI, heart failure, valvular heart disease, dilated cardiomyopathy, malignant tumor, advanced liver disease, renal failure, autoimmune diseases, and other inflammatory diseases and women who were pregnant and breast-feeding were excluded from the study. Control subjects (*n* = 20) were healthy volunteers, recruited from the same population and the same area of China as the patients' group. Total RNAs isolated from 5 BSS of UA patients and 5 healthy volunteers were used for gene expression oligonucleotide microarray and microRNA microarray. The results obtained from bioinformatics analysis of microarray were then prospectively tested in a validation cohort of 30 BSS of UA patients, 30 non-BSS of UA patients, and 15 healthy volunteers. The study complied with the Declaration of Helsinki and was approved by the local ethics committee. All individuals gave their written informed consent to participate in the study.

### 2.2. Plasma Collection and RNA Isolation

Whole blood samples (10 mL) were drawn from each of the 85 participants (35 UA patients with BSS, 30 non-BSS of UA patients and 20 healthy volunteers) with 19-gauge needle for clean venipuncture of an antecubital vein on the following morning after arrival. Timing of phlebotomy of UA patients compared with onset of chest pain was <24 h. Blood was drawn into EDTA-containing tubes and PBMCs were isolated by performing density gradient centrifugation with Ficoll (Invitrogen, Carlsbad, CA, USA). Total RNAs were extracted from PBMCs using using Trizol reagent (Invitrogen) according to the manufacturer's instruction. RNA quantity and purity were assessed using NanoDrop ND-1000 (Thermo Scientific, Waltham, MA, USA). Pass criteria for absorbance ratios were established at A260/A280 ≥ 1.8 and A260/A230 ≥ 1.5 indicating acceptable RNA purity. RNA Integrity Number (RIN) values were ascertained using Agilent RNA 6000 Nano assay (Agilent Technologies, Santa Clara, CA, USA). Pass criteria for RIN value were established at ≥6 indicating acceptable RNA integrity. Genomic DNA contamination was evaluated by gel electrophoresis. The RNA samples were stored at −80°C until analysis.

### 2.3. mRNA Expression Profiling

Total RNAs of 10 participants (5 UA patients with BSS and 5 healthy volunteers) were used for mRNA expression profiling by the Human Whole Genome OneArray v5 (Phalanx Biotech Group, Hsinchu, Taiwan). It contained 30275 DNA oligonucleotide probes, and each probe was a 60-mer designed in the sense direction. Among the probes, 29187 probes corresponded to the annotated genes in RefSeq v38 and Ensembl v56 database. Besides, 1088 control probes were also included. Fluorescent aRNA targets were prepared from 1 or 2.5 *μ*g total RNA samples using OneArray Amino Allyl aRNA Amplification Kit (Phalanx Biotech Group) and Cy5 dyes (Amersham Pharmacia, Piscataway, NJ, USA). Fluorescent targets were hybridized to the Human Whole Genome OneArray with Phalanx hybridization buffer using Phalanx Hybridization System. After 16 hrs hybridization at 50°C, nonspecific binding targets were washed away by three different washing steps (Wash I 42°C 5 minutes; Wash II 42°C 5 minutes, 25°C 5 minutes; Wash III rinse 20 times), and the slides were dried by centrifugation and scanned by Axon 4000B scanner (Molecular Devices, Sunnyvale, CA, USA). The intensities of each probe were obtained by GenePix 4.1 software (Molecular Devices). The raw intensity of each spot was loaded into Rosetta Resolver System (Rosetta Biosoftware, Seattle, WA, USA) to process data analysis. The error model of Rosetta Resolver System could remove both systematic and random errors form the data. Those probes with background signals were filtered out. Probes that passed the criteria were normalized by 50% median scaling normalization method. Normalized spot intensities were transformed to mRNA expression log_2_ ratios between the UA patients with BSS and healthy controls. 

### 2.4. MicroRNA Expression Profiling

We performed miRNA expression profiling in the same set of samples (5 UA patients with BSS and 5 healthy volunteers) that were used in the analysis of mRNA microarray. MiRNA microarray analysis was performed by using the Human miRNA OneArray v4 (Phalanx Biotech Group). It contained triplicated 1884 unique miRNA probes from Human (miRBase Release v18) each printed in technical triplicate, and 144 experimental control probes. Small RNA was preenriched by Nanoseplook (Pall Corporation, Port Washington, NY, USA) from 2.5 *μ*g total RNA samples and labeled with miRNA ULS Labeling Kit (Kreatech Diagnostics, Vierweg, Amsterdam, The Netherlands). Labeled miRNA targets were hybridized to the Human miRNA OneArray v4 with OneArray Hybridization System. After 16 hrs hybridization at 37°C, nonspecific binding targets were washed away by three different washing steps (Wash I 37°C 5 minutes; Wash II 37°C 5 minutes, 25°C 5 minutes; Wash III rinse 20 times), and the slides were dried by centrifugation and scanned by an Axon 4000B scanner (Molecular Devices). The Cy5 fluorescent intensities of each probe were analyzed by GenePix 4.1 software (Molecular Devices). The raw intensity of each probe was processed by R program. Probes that passed the criteria were normalized by 75% median scaling normalization method. Normalized spot intensities were transformed to miRNA expression log_2_ ratios between the UA patients with BSS and healthy controls. 

### 2.5. Integrated Bioinformatics Analysis of the mRNA and MicroRNA Expression Profiles

#### 2.5.1. Identification of Differentially Expressed mRNAs and miRNAs

The expressions of mRNAs with log_2_ ratio ≥ 1 or log_2_ ratio ≤ −1 and *P* value < 0.05 were defined as differential mRNAs. The expressions of miRNAs with log_2_ ratio ≥ 0.8 or log_2_ ratio ≤ −0.8 and *P* value < 0.05 were defined as differential miRNAs. Hierarchical clustering analysis combined with a heatmap was applied to evaluate the overall reproducibility and variation of  5 samples within each group and the differences between the 2 groups. An average linkage hierarchical clustering was performed with clustering software Cluster3.0 and Java TreeView-1.1.6r2 was applied to generate the heatmap [28, 29]. 

#### 2.5.2. KEGG Pathway Analysis Using DAVID

There were thousands of deregulated genes; we cannot analyze the function of all these deregulated genes one by one, so we could only focus on the main functions of deregulated genes. We used DAVID Bioinformatics Resources 6.7 (the Database for Annotation, Visualization, and Integrated Discovery) [30], a comprehensive set of functional annotation tools for understanding the biological meaning behind large lists of genes, to identify enriched KEGG (Kyoto Encyclopedia of Genes and Genomes) pathways [31] information for differential mRNAs between UA patients with BSS and healthy controls. EASE Score, a modified Fisher Exact *P* Value, was used to measure the gene enrichment in annotation pathways and Benjamini-Hochberg False Discovery Rate was used as testing correction. The threshold of EASE Score was set 0.1. Because miRNAs were usually negatively correlated with target mRNAs and differentially expressed miRNAs were mainly upregulated, pathways enriched of downregulated mRNAs were chose for further analysis and downregulated mRNAs were marked in green on these pathways by Search & Color Pathway in KEGG [31]. MiRNAs which could regulate downregulated genes of these pathways were predicted by one of the 2 algorithms of DIANA-microT and Targetscan [32, 33]. If these predicted miRNAs were among the actually upregulated miRNAs, they were marked in red on these pathways.

#### 2.5.3. Integrated miRNA/mRNA Network Analysis

MiRNAs were usually negatively correlated with target mRNAs and differentially expressed miRNAs in BBS with UA group were mainly upregulated, so upregulated miRNAs and downregulated mRNAs were uploaded into the miRTrail, a knowledge-based tool for integrative network analysis that allowed for studying the interactions between microRNAs and their target mRNAs [34]. For each upregulated miRNA, the target mRNAs were predicted by the respective web-resource microCosm and the prediction threshold was set at 0.01. The predicted target mRNAs were compared with the actually downregulated mRNAs in order to find the overlap between them. The miRNAs that could regulate these overlap mRNAs were ranked based on the number of deregulated target mRNAs and the top 5% were selected. The interactive network of selected miRNAs and actually deregulated target mRNAs was visualized by the network analyzers and viewers BiNA [35]. 

#### 2.5.4. Protein-Protein Interactions Network Analysis

In the integrated miRNA/mRNA network, there were hundreds of upregulated mRNAs. To further understand the function of these miRNAs, we use a method of combing the protein-protein interactions (PPIs) network and hubs. Protein-Protein Interactions (PPIs) were commonly understood as physical contacts with molecular docking between proteins that occur in a cell or in a living organism *in vivo* [36]. Each of these interactions was specifically adapted to carry out certain biological functions. A PPIs network was represented as proteins as nodes and interactions between nodes are edges. To further understand the function of the upregulated miRNAs, a PPIs network for mRNAs in the miRNA/mRNA interactive network was built by Reactome FI, a Cytoscape plugin [37]. This plugin accessed the Reactome Functional Interaction (FI) network, a highly reliable, manually curated pathway-based protein functional interaction network covering close to 50% of human proteins, and allowed you to construct an FI subnetwork based on a set of genes by using linker genes. The PPIs network was visualized using the Cytoscape software [38].

Hubs were highly connected nodes in a network and vital for the proper function of a network [39]. CytoHubba, a Cytoscape plugin, was used to find the hubs of the PPIs network [40]. It evaluated node essentiality by topological characters. The degree of a node was the number of links incident to this node in a network and it was chose for topological analysis in the study. Top 10 essential nodes ranked by degree scores were selected as hubs from the network.

### 2.6. Reverse Transcription and qRT-PCR of miRNA and mRNA

Combining pathway analysis with network analysis, hsa-miR-146b-5p and has-miR-199a-5p seemed to be the most involved miRNAs in UA patients with BSS. Thus, to support the robustness of our analysis, differences in expression of the 2 miRNAs and 2 downregulated target mRNAs (CALR and TP53) were validated in an independent cohort of 30 UA patients with BSS, 30 non-BSS of UA patients, and 15 healthy controls by qRT-PCR. To assay for miRNAs, 1 *μ*g of purified total RNA generated cDNA using the QuantiMir RT Kit, following the manufacturer's instructions (System Biosciences, Mountain View, CA, USA). qRT-PCR was performed using 2X SYBR Green qPCR Mastermix (Roche Applied Science, Indianapolis, IN, USA) 7900HT Fast Real-Time PCR System (Applied Biosystems, Carlsbad, CA, USA). The mature sequences of hsa-miR-146b-5p and has-miR-199a-5p were used as the forward primers, and the 3′ universal reverse primer provided from the QuantiMir RT Kit (System Biosciences) was used as the reverse primer. The human U6 RNA was amplified in parallel as the internal control. All the miRNA forward primer sequences were listed in Table 2. The Mastermix contents included 10 *μ*L 2X SYBR Green qPCR Mastermix buffer, 2 *μ*L miRNA-specific forward primer, 1 *μ*L universal reverse primer, 1 *μ*L diluted QuantiMir cDNA and 6 *μ*L RNase-free water. The thermal cycling conditions were at 95°C for 5 minutes, followed by 40 cycles of at 95°C for 30 seconds, at 55°C for 30 seconds, 72°C for 50 seconds, and a final extension at 72°C for 8 minutes. For mRNA expression analysis, 2 *μ*g of purified total RNA generated cDNA using RevertAid First Strand cDNA Synthesis Kit, following the manufacturer's instructions (Thermo Scientific). qRT-PCR was performed using 2X SYBR Green qPCR Mastermix (Roche Applied Science) 7900HT Fast Real-Time PCR System (Applied Biosystems). GAPDH was used as the internal control. All the mRNA primer sequences were listed in Table 3. The Mastermix contents included 10 *μ*L 2X SYBR Green qPCR Mastermix buffer, 1 *μ*L mRNA-specific forward primer, 1 *μ*L mRNA-specific reverse primer, 1 *μ*L cDNA, and 7 *μ*L RNase-free water. The thermal cycling conditions were at 95°C for 5 minutes, followed by 35 cycles of at 95°C for 35 seconds, at 54°C for 35 seconds, 72°C for 50 seconds and a final extension at 72°C for 8 minutes. The reaction products were analyzed by electrophoresis in 3% agarose gels to confirm specificity. Analysis was performed by relative standard curve method for quantification [41]. 

### 2.7. Statistics

All results for continuous variables were expressed as means ± SEM, if not stated otherwise. For group-wise comparisons, Man-Whitney test (2 groups), ANOVA, Kruskal-Wallis test (*n* groups), or Student's *t*-test (2 groups) were used as appropriate. For categorical variables, the Chi-square test or Fischer's exact test was used. All tests were performed 2-sided and a significance level of *P* < 0.05 was considered to indicate statistical significance. For all statistical analyses, the statistical software SPSS 16.0 (Statistical Package for the Social Sciences, Chicago, IL, USA) for Windows was used. GraphPad Prism 5 (GraphPad software, San Diego, CA, USA) was used to draw bar and box chars.

## 3. Results

### 3.1. Basic Clinical Characteristics of Subjects

A total of 85 subjects were studied. 35 UA patients with BSS and 30 UA patients with non-BSS had angiographically documented CAD. 20 healthy volunteers were selected as the healthy controls. 5 UA patients with BSS and 5 healthy volunteers were used for mRNA and miRNA microarray analysis. The clinical characteristics of the 2 groups of microarray analysis were summarized in Table 4. 30 UA patients with BSS, 30 UA patients with non-BSS and 15 healthy volunteers were used for qRT-PCR. The clinical characteristics of the 3 groups of qRT-PCR were summarized in Table 5. There were no significant differences in age, percentage of males, BMI (body mass index), percentage of active smoker, history of type 2 diabetes mellitus, total cholesterol, LDL cholesterol, HDL cholesterol, triglycerides, and calcium-channel blockers medication between UA patients with BSS and healthy control group in microarray analysis. There were significant differences in other clinical parameters between the two groups in microarray analysis (*P* < 0.05). There were no significant differences in age, percentage of males, BMI (body mass index), percentage of active smoker, total cholesterol, LDL cholesterol and CRP among UA patients with BSS, 30 UA patients with non-BSS and healthy control group in qRT-PCR analysis. There were significant differences in other clinical parameters among the 3 groups in qRT-PCR analysis (*P* < 0.05). There were no significant differences in HDL cholesterol, triglycerides (TG), hypertension, concurrent medication, and number of vessels between UA patients with BSS and UA patients with non-BSS in qRT-PCR analysis. There was significant difference in BSS grades between UA patients with BSS and UA patients with non-BSS in qRT-PCR analysis (*P* < 0.05).

### 3.2. Identification of Differentially Expressed mRNAs and miRNAs

**Figure 1:**
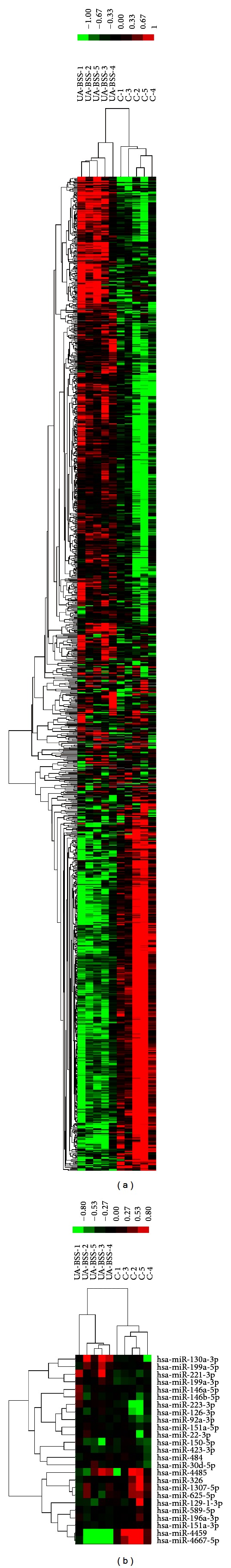
Heat maps of mRNAs and miRNAs differentially expressed between UA patients with BSS and healthy controls. (a) Heat map and cluster analysis of the 1,206 differentially expressed probes between UA patients with BSS and healthy control. Red and green represented, respectively, differentially upregulated and downregulated mRNAs in UA patients with BSS. (b) Heat map and cluster analysis of the 25 differentially expressed miRNAs between UA patients with BSS and healthy control. Red and green represented, respectively, differentially upregulated and downregulatedmiRNAs in UA patients with BSS. Gray was for missing values. UA-BSS: unstable angina patients with Blood stasis syndrome; C: control group.

A list of 1081 mRNAs was identified as differentially expressed between UA patients with BSS and the healthy controls (Figure 1(a)): 673 (56%) overexpressed and 408 (44%) underexpressed. The 1081 mRNAs were corresponding to 1206 nonunique probes because multiple probes in the microarray platform could be representative of a single mRNA. A list of 25 miRNAs was identified as differentially expressed between UA patients with BSS and the healthy control (Figure 1(b), Table 6): 23 (92%) overexpressed and 2 (8%) underexpressed.

### 3.3. Pathway Analysis Using DAVID

**Figure 2:**
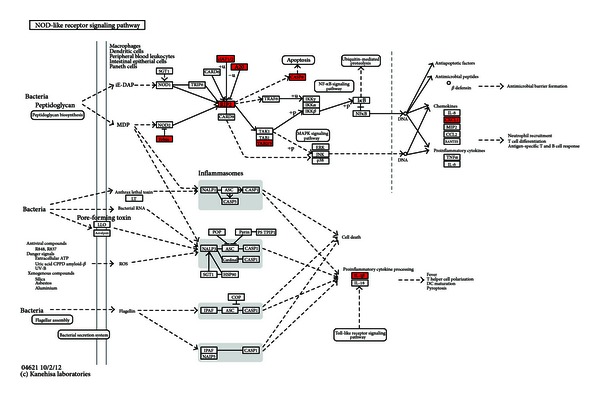
NOD-like receptor signaling pathway. Upregulated mRNAs were depicted in red by Search & Color Pathway in KEGG.

**Figure 3:**
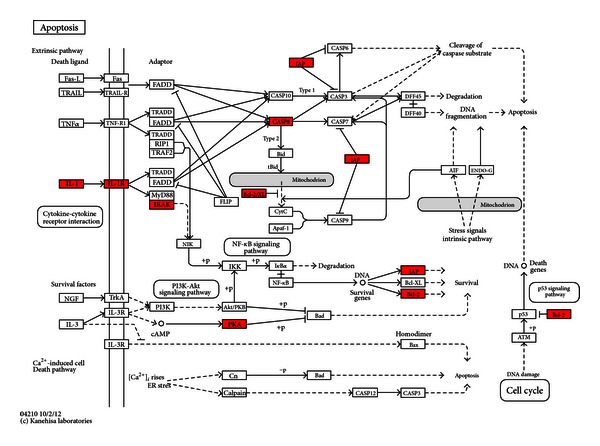
Apoptosis pathway. Upregulated mRNAs were depicted in red by Search & Color Pathway in KEGG.

**Figure 4:**
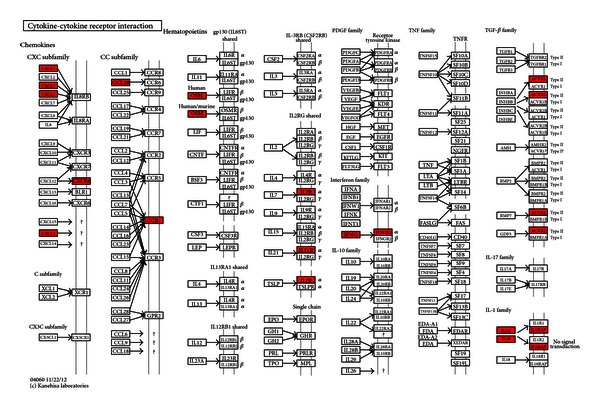
Cytokine-cytokine receptor interaction pathway. Upregulated mRNAs were depicted in red by Search & Color Pathway in KEGG.

**Figure 5:**
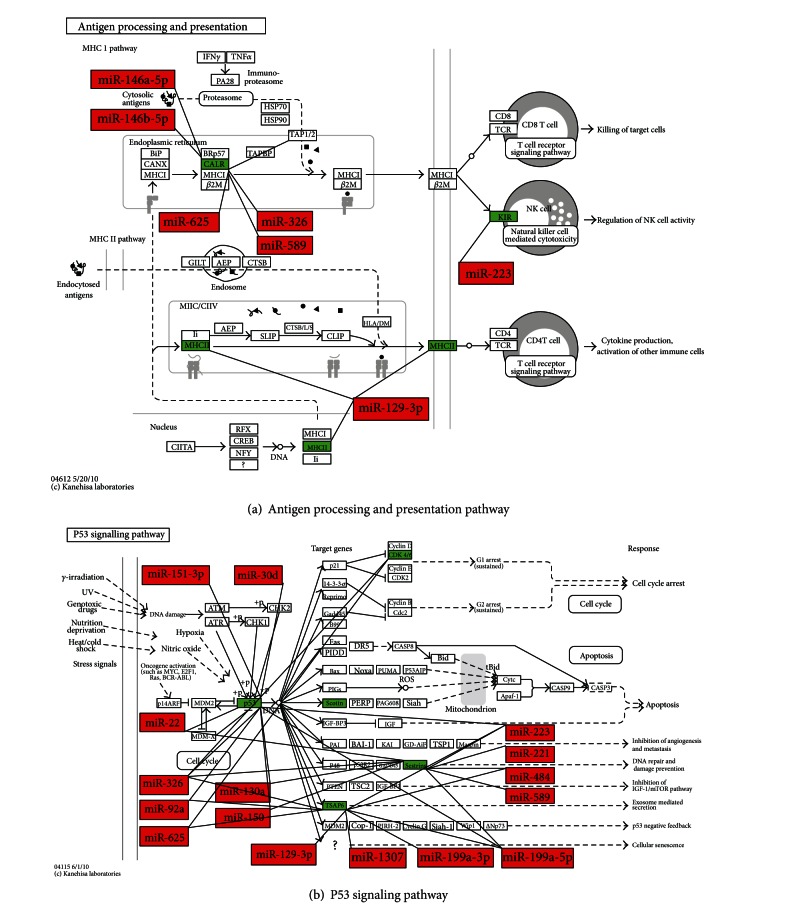
The regulation of upregulated miRNAs on the downregulated mRNAs in antigen processing and presentation pathway and p53 signaling pathway. Upregulated miRNAs were depicted in red and downregulated mRNAs were depicted in green on the maps.

The KEGG pathway analysis of upregulated and downregulated mRNAs between UA patients with BSS and the healthy controls using DAVID was shown in Tables 7 and 8. Upregulated genes were enriched in 7 pathways and downregulated genes were enriched in 6 pathways. Among 7 pathways enriched of upregulated genes, NOD-like receptor signaling pathway, apoptosis pathway, and cytokine-cytokine receptor interaction pathway were closely related with UA. In the NOD-like receptor signaling pathway, ERBB2IP, BIRC2, BIRC3, TNFAIP3, RIPK2, CASP8, TAB2, CXCL1 and IL1B were upregulated. (Figure 2) In the apoptosis pathway, IL1A, IL1B, IL1RAP, IRAK3, PRKAR2B, BIRC2, BIRC3, BCL2, and CASP8 were upregulated. (Figure 3) In the cytokine-cytokine receptor interaction pathway, IL1A, IL1B, IL1RAP, IL7R, IL21R, CCR1, CXCR4, CXCL1, CXCL3, CXCL5, PF4V1,CCL20, IFNG, IFNGR1, OSM and ACVR2A were upregulated. (Figure 4) Among 6 pathways enriched of downregulated genes, the antigen processing and presentation pathway and p53 signaling pathway were closely related with UA, so the 2 pathways were chose for next analysis. Upregulated miRNAs which could regulate downregulated genes of the 2 pathways were analyzed by one of the 2 algorithms of DIANAmT and Targetscan and mapped on the 2 pathways. In the antigen processing and presentation pathway, CALR, HLA-DRB1, HLA-DRB5, KLRC1, KLRC3, KIR3DS1 and KIR2DS3 were downregulated. CALR could be the target gene of miR-146a-5p, miR-146b-5p, miR-326, miR-589 and miR-625. HLA-DRB1 and HLA-DRB5 could be the target gene of miR-129-3p. KLRC1, KLRC3, KIR3DS1 and KIR2DS3 could be the target gene of miR-223. (Figure 5(a)) In the p53 signaling pathway, TP53, CDK4, STEAP3, SHISA5 and SESN2 were downregulated. TP53 could be the target gene of miR-129-3p, miR-130a, miR-1307, miR-151-3p, miR-199a-3p, miR-199a-5p, miR-22, miR-221, miR-223, miR-30d, miR-326, miR-484, miR-589, miR-625 and miR-92a. CDK4 could be the target gene of miR-326 and miR-625. STEAP3 could be the target gene of miR-129-3p, miR-1307, miR-199a-3p, miR-199a-5p, miR-223, miR-326, miR-625 and miR-92a. SESN2 could be the target gene of miR-130a, miR-150, miR-199a-5p, miR-22, miR-221, miR-223, miR-326, miR-484 and miR-589 (Figure 5(b)).

### 3.4. Integrated miRNA/mRNA Network Analysis

**Figure 6:**
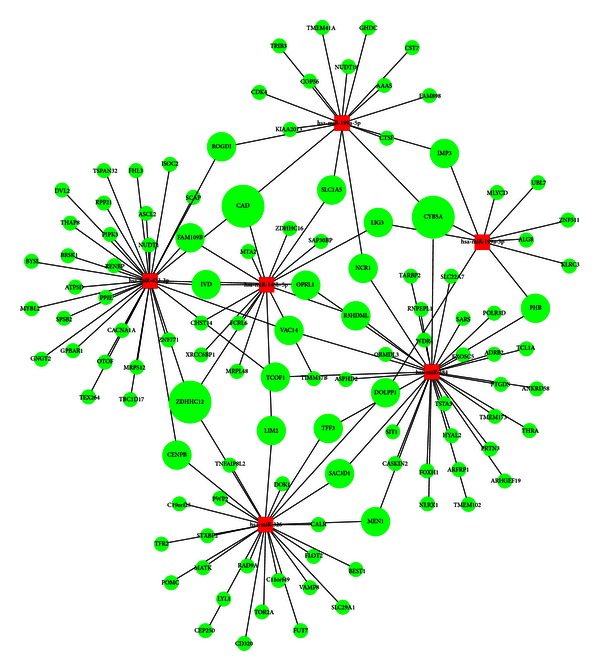
The miRNA/mRNA interactive network. Round represented mRNAs and rectangular represented miRNAs. Red color indicated upregulation and green color downregulation, respectively. Sizes of the mRNAs were according to their degrees.

23 upregulated miRNAs and 408 downregulated mRNAs were uploaded into miRTrail. 4250 target mRNAs were predicted by microCosm. 115 mRNAs were found to be the overlap between the predicted target mRNAs and the actually downregulated mRNAs. 6 upregulated miRNAs were selected according to the top 5% of the rank based on the number of deregulated target mRNAs. The interactive network of 115 downregulated mRNAs and 6 upregulated miRNAs was visualized by BiNA. The 6 upregulated miRNAs were miR-146b-5p, miR-199a-3p, miR-199a-5p, miR-326, miR-423-3p and miR-484 (Figure 6). 

### 3.5. Protein-Protein Interactions Network Analysis

**Figure 7:**
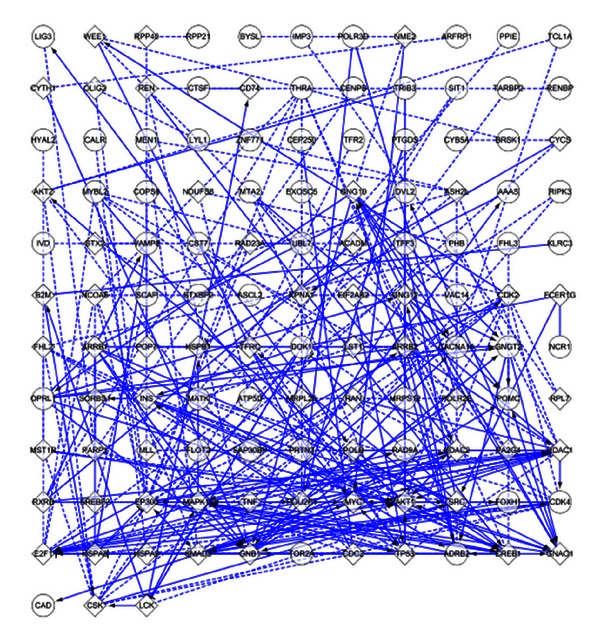
The PPIs network of downregulated mRNAs of the miRNA/mRNA interactive network. Rectangular represented downregulated mRNAs and round represented added interactive mRNAs. The interactions between two mRNAs extracted from pathways were shown as solid lines while those predicted interactions were shown as dashed lines. Extracted interactions involved in activation, expression regulation, or catalysis were shown with an arrowhead on the end of the line, while interactions involved in inhibition were shown with a “T” bar.

**Figure 8:**
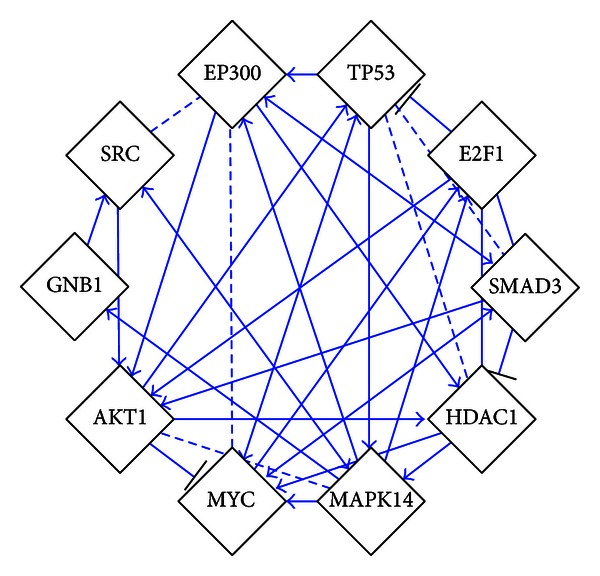
Hubs of the PPIs network.

Based on the integrated miRNA/mRNA network analysis results, a PPIs network for 115 downregulated mRNAs was built by Reactome FI plugin (Figure 7). It included 124 nodes and 291 edges. Hubs of the PPIs network were found by CytoHubba. Hubs were MAPK14, AKT1, EP300, HDAC1, TP53, E2F1, SMAD3, GNB1, MYC, and SRC (Figure 8). The degrees of hubs were listed in Table 9.

### 3.6. qRT-PCR of miRNA and mRNA

**Figure 9:**
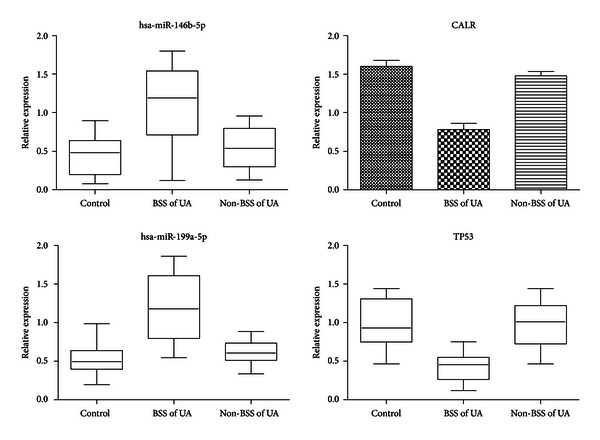
Validation of differentially expressed miRNAs and mRNA among UA patients with BSS, UA patients with non-BSS, and healthy control by RT-PCR analysis.

Representative qRT-PCR results for hsa-miR-146b-5p, has-miR-199a-5p, CALR, and TP53 were reported in Figure 9. Hsa-miR-146b-5p and has-miR-199a-5p were upregulated, while CALR and TP53 were downregulated in UA patients with BSS compared to UA patients with non-BSS or the healthy control (*P* < 0.05). These results confirmed our bioinformatics analysis. 

## 4. Discussion

In this study, using a systems biology approach we exploited critically deregulated miRNAs and mRNAs involved in pathogenesis of BSS of UA and potential biomarkers for BSS of UA. Our integration strategy started by extracting differentially expressed mRNAs and miRNAs between UA patients with BSS and the healthy control from the microarray analysis and then took them to integrative bioinformatics analysis. The robustness of our analysis was confirmed by qRT-PCR.

### 4.1. mRNA Expression Data

Regarding thousands of deregulated genes, we cannot analyze the function of all these deregulated genes one by one, so we only focused on the function of deregulated genes enriched in KEGG pathways. The function of deregulated genes in the pathways related with UA would be discussed in the following part to understand the biomedical mechanisms of BSS of UA. 

Among upregulated genes of NOD-like receptor signaling pathway (Figure 2), RIPK2 was a key mediator of the activation of apoptosis pathway [42]. Apoptosis was known to occur in advanced plaques at higher levels, comparing both patients with stable angina and control subjects [43, 44]. The apoptosis of vascular smooth muscle cells (VSMCs) and macrophages altered plaque composition and made it prone to disruption and acute luminal thrombosis [45, 46]. CASP8, an important executor of apoptosis [47], was upregulated in NOD-like receptor signaling pathway. The upregulation of CASP8 indicated active apoptosis in BSS of UA. RIPK2 was critical for both the innate and adaptive immune pathways [48]. However, there were fewer studies about the role of RIPK2 in UA. Our study showed that RIPK2 may contribute to the apoptosis in BSS of UA by activating CASP8. This indicated the innate and adaptive immunity may regulate the apoptosis in BSS of UA. Except RIPK2, CXCL1, and IL1B were also key upregulated genes in the NOD-like receptor signaling pathway. Studies showed that CXCL1 could support arrest of human monocytic cell lines and primary monocytes under flow conditions and may play an important role in monocyte recruitment to atherosclerotic lesions [49]. IL-1B was a proinflammatory cytokine with pleiotropic effects implicated in the various stages of atherosclerosis [50, 51]. Studies showed that IL-1B was at higher levels in UA patients, comparing both stable angina ones and control subjects in peripheral blood [52, 53]. Both CXCL1 and IL1B were important proinflammatory mediators, and they promoted the inflammation. Inflammation can regulate the integrity of the interstitial collagen of the plaque's fibrous cap and be responsible for plaque rupture and triggering UA [54]. The upregulation of CXCL1 and IL1B may promote inflammation in BSS of UA. In the apoptosis pathway, the effect of upregulation of IL1B and CASP8 was discussed. IL1A, IL1B, and IL1RAP all belonged to the IL-1 family. IL-1 was proinflammatory and destabilized the proteinaceous scaffold of the cap by inducing up-regulation of matrix metalloproteinases [55]. The upregulation of IL-1 and IL1RAP may promote the inflammation in BSS of UA. IRAK3 was a kinase-deficient member of the TLR/IRAK family that was an important negative regulator of TLR signaling and regulated innate immune homeostasis [56]. The latest research showed that IRAK3 was a key inhibitor of TLR2/NF-*κ*B mediated chronic inflammation [57]. Although the role of IRAK3 in UA was not defined, our study showed that the upregulation of IL-1 may activate IRAK3 to regulate innate immunity and inhibit inflammation. PRKAR2B was one of the regulatory subunits bound to cAMP. The activation of cAMP/PKA pathway induced the phosphorylation and inactivation of BAD, a proapoptotic protein [58]. BIRC2 and BIRC3 were members of the inhibitor of apoptosis protein (IAP) family [59]. BCL2 was an antideath factor, and it inhibited the apoptosis of VSMCs and macrophages in advanced atherosclerotic lesions [60]. The upregulation of PRKAR2B, BIRC2, BIRC3, and BCL2 may suppress the apoptosis in BSS of UA. In the cytokine-cytokine receptor interaction pathway, the upregulation of IL1A, IL1B, IL1RAP and IL7R promoted the inflammation in UA. Although there was lack of investigations about the role of IL21R in UA, studies demonstrated that IL21R involved in chronic inflammation [61]. The upregulation of IL21R may cooperate with other upregulated interleukins to exacerbate the inflammation in BSS of UA. CCR1, CXCR4, CXCL1, CXCL3, CXCL5, PF4V1, and CCL20 were chemokines and their receptors which were critical for the recruitment of effector immune cells to the site of inflammation [62]. CCR1 and CXCR4 involved in both leukocyte homeostasis and inflammation [63, 64]. The expressed mRNA levels of CCR1 and CXCR4 were higher in PBMCs from UA patients, comparing both stable angina ones and control subjects [65]. CXCL1 and CXCL3 acted as arrest chemokines for monocyte adhesion on vascular cell adhesion molecule (VCAM)-1 under flow in the presence of  P-selectin [66]. CXCL5 was demonstrated to attract neutrophils [67]. CCL20 was demonstrated in plaque initiation [68]. IFNG was found to be highly produced by CD4^+^ T cells within human coronary plaques [69]. Monocytes from UA patients exhibited a molecular fingerprint of recent IFN-g triggering [70]. OSM was a member of the interleukin (IL)-6 superfamily cytokines. OSM was demonstrated to contribute to plaque destabilization by inducing vascularization of the lesion due to its angiogenic properties and involving in plaque thrombogenicity [71]. Taken together, the upregulation of these cytokines and their receptors may promote the inflammation and destabilize the plaque in BSS of UA.

In the antigen processing and presentation pathway, CALR (calreticulin) was an ER (endoplasmic reticulum) luminal Ca^2+^-buffering chaperone, and involved in regulation of intracellular Ca^2+^ homoeostasis and ER Ca^2+^ capacity [72]. Recent studies showed that enhanced CALR expression in mature cardiomyocytes disrupted intracellular calcium regulation, leading to calcium-dependent apoptosis [73]. It was reported that fibroblast growth factor 2(FGF-2) induced angiogenesis after sustained ischemia with downregulation of CALR expression in myocardium and the CALR expression was negatively correlated to angiogenesis [74]. In addition, the binding of CALR to CD91 stimulated proinflammatory responses [75]. The downregulation of CALR may attenuate apoptosis and inflammation, and promote angiogenesis in BSS of UA. HLA-DRB1 and HLA-DRB5 belonged to the HLA class II molecule which played a central role in the immune system by presenting peptides derived from extracellular proteins [76]. There was a marked upregulation of HLA class II in unstable plaques and HLA class II may contribute to the T-cell response against antigens in the unstable plaques [77]. The downregulation of HLA class II may inhibit the T-cell response in plaques in BSS of UA. KLRC1 and KLRC3 were members of natural killer (NK) cell lectin-like receptors subfamily C. KIR3DS1 and KIR2DS3 were members of natural killer (NK) cell immunoglobulin-like receptors. These NK cell receptors played an important role in regulation of the immune response [78]. NK cells were identified in human and mouse atherosclerotic lesions and infiltrated the vessel wall and promoted atherosclerotic lesion development [79]. The downregulation of these NK cell receptors may indicate the suppression of immune response mediated by NK cells in BBS of UA. In the p53 signaling pathway, TP53 (p53) responded to diverse cellular stresses to regulate target genes that induced cell cycle arrest, apoptosis, senescence, DNA repair, or changes in metabolism [80]. The increased TP53 expression was related with the enlargement of necrotic cores, plaque rupture, and clinical manifestations of carotid plaques. Concomitant increases of TP53 level may lead to the apoptosis and atheroma progression in patients with carotid atherosclerosis [81]. The downregulation of TP53 may inhibit the apoptosis in BSS of UA. CDK4 was a catalytic subunit of the cyclin-dependent kinase. In proliferating cells, the activation of CDK4 was necessary for cell cycle progression [82]. Proliferation of VSMCs and macrophages was observed in atherosclerotic plaques [83]. There was evidence that oxLDL-stimulated VSMC proliferation was associated with significant increases in the expression of CDK4 [84]. The downregulation of  CDK4 may inhibit cell proliferation in BSS of UA. STEAP3 regulated apoptosis and the cell cycle [85]. SHISA5 encoded a protein named Scotin. Scotin induced apoptosis by causing cell cycle arrest [86]. SESN2 played a role in the regulation of cell growth and survival [87]. There was still lack of research about the function of STEAP3, SHISA5, and SESN2 in UA. Whether these genes involved in the regulation of apoptosis of BSS in UA was worth further exploring.

Putting the functional analysis of pathways together, most of the upregulated genes in BSS of UA were implicated in inflammation, apoptosis, and innate and adaptive immunity, and most of the downregulated genes in BSS of UA were implicated in apoptosis, angiogenesis, inflammation, immune response, and cell cycle arrest. The inflammation and immune response in BSS of UA was in accordance with previous reports demonstrating inflammatory and immune related genes were related to BSS of CAD by microarray analysis [88]. In addition, the analysis showed that apoptosis was involved in BSS of UA and some genes related with the regulation of cell cycle may regulate apoptosis in BSS of UA. Apoptosis added a new layer for the complex pathophysiology of BSS of UA. Interestingly, we found that some genes of the active innate and adaptive immunity may regulate inflammation and apoptosis in BSS of UA, such as RIPK2 and IRAK3. The deregulation and imbalance of apoptosis, inflammation and immunity could be the key biomedical mechanisms in BSS of UA. 

### 4.2. miRNA Expression Data

Compared with healthy controls, the miRNA expression of BSS of UA was mainly upregulated. Considering the negative regulation of miRNA on gene, it indicated that miRNAs may mainly play an inhibitory role in BSS of UA. Among the upregulated miRNAs, miR-126, miR-129, miR-146, miR-150, miR-151a-3p, miR-199a-5p, miR-221, miR-223, miR-30d, miR-326 and miR-92a were reported to be upregulated in PBMCs of CAD patients compared with healthy controls [89–91]. There were compelling evidences that some of these upregulated miRNAs played fundamental roles in the development and progression of UA. miR-126 was an endothelial cell-restricted microRNA and enhanced the proangiogenic actions of VEGF and FGF and promoted blood vessel formation [92]. miR-130a downregulated the antiangiogenic homeobox proteins GAX and HoxA5 [93]. The proangiogenic properties of miR-126 and miR-130a suggested that their upregulation may enhance angiogenesis in BSS of UA. In human blood cells, miR-150 was selectively packaged into microvesicles (MVs) and actively secreted. Secreted monocytic miR-150 enhanced targeted endothelial cell migration. MVs isolated from the plasma of patients with atherosclerosis contained higher levels of miR-150, and they more effectively promoted endothelial cell migration than MVs from healthy donors [94]. The upregulation of miR-150 may promote the endothelial cell migration in BSS of UA. miR-221 was necessary for VSMC proliferation and vascular neointimal lesion formation [95]. The upregulation of miR-221 may promote VSMC proliferation in BSS of UA. miR-92a was highly expressed in endothelial cells and regulated angiogenic functions of endothelial cells. Forced overexpression of miR-92a in endothelial cells blocked angiogenesis *in vitro* and *in vivo *[96]. Upregulation of miR-92a may hinder angiogenesis in BSS of UA. 

### 4.3. Integrated miRNA/mRNA Netwok Analysis

Among upregulated miRNAs, miR-146b-5p, miR-199a-3p, miR-199a-5p, miR-326, miR-423-3p, and miR-484 were found to be key deregulated miRNAs in miRNA/mRNA network. MAPK14, AKT1, EP300, HDAC1, TP53, E2F1, SMAD3, GNB1, MYC, and SRC were found to be hubs of the PPIs network of downregulated target genes of the 6 miRNAs. Among the hubs, EP300, HDAC1, TP53, E2F1, SMAD3, and MYC were involved in the regulation of cell cycle [80, 97–101]. AKT1 and TP53 were involved in the regulation of apoptosis [80, 102]. MAPK14, AKT1, and SRC were involved in the regulation of angiogenesis [103–105]. It indicated that downregulated target genes of the 6 miRNAs were mainly functioned in the regulation of cell cycle, apoptosis, and angiogenesis. This result was partly consistent with the pathway analysis of downregulated genes. Interestingly, TP53 was confirmed by both pathway and network analysis to be the key downregulated target genes.

Compared the network analysis with pathway analysis, we chose miR-146b-5p and miR-199a-5p and their target genes for further analysis for 2 reasons. First, miR-146b-5p and miR-199a-5p were proved to be key upregulated miRNAs in both pathway and network analysis. Second, CALR and TP53 were the key downregulated genes in BSS of UA, while CALR was the target gene of miR-146a-5p and TP53 was the target gene of miR-199a-5p. Although there was lack of research about the role of miR-146b-5p in UA, miR-146b was demonstrated to be rapidly induced in human monocytes in response to a variety of microbial components and proinflammatory cytokines [106]. It was reported that miR-146b decreased the expression of TNF*α*, IL-1B, and IL-6 in THP-1 monocytes [106, 107]. Other studies showed that IL-1 receptor signaling initiated both miR-146b upregulation and cytokine secretion, and that miR-146b was expressed in response to rising inflammatory cytokine levels and suppressed IL-6 and IL-8 secretion in primary human fibroblasts [108]. Moreover, miR-146b was induced by the potent proresolving mediator RvD1 in human macrophages in the resolution of inflammation and decreased protein levels of proinflammatory IL-8 and RANTES [109]. Furthermore, a recent study showed that miR-146b-5p, decreased in monocytes during obesity, was a major mediator of the anti-inflammatory action of globular adiponectin [110]. Collectively, all these studies implied that miR-146b-5p was induced in the inflammation and played an anti-inflammatory role in innate immunity. According to the pathway analysis, CALR could be the target gene of miR-146b-5p and upregulation of miR-146b-5p may induce the downregulation of CALR. According to functional analysis, both upregulation of miR-146b-5p and downregulation of CALR could attenuate the inflammation, and it implied that miR-146b-5p may attenuate inflammation by targeted repression of CALR in BSS of UA. Moreover, if CALR was confirmed to be the target gene of miR-146b-5p, miR-146b-5p may also function to inhibit apoptosis and promote angiogenesis by suppressing CALR in BSS of UA. miR-199a was reported to be acutely downregulated in cardiomyocytes in hypoxia and replenishing miR-199a during hypoxia reduced apoptosis [111]. According to the pathway analysis, TP53 could be the target gene of miR-199a-5p and upregulation of miR-199a-5p may induce the downregulation of TP53. According to functional analysis, both upregulation of miR-199a-5p and downregulation of TP53 could inhibit apoptosis, and it implied that miR-199a-5p may inhibit apoptosis by targeted repression of TP53 in BSS of UA.

Based on functional enrichment analysis of upregulated genes, there was active inflammation, apoptosis, and immune response in BSS of UA. Intriguingly, upregulation of miR-146b-5p and miR-199a-5p may extenuate inflammation and apoptosis in BSS of UA. Analysis of miR-146b-5p and miR-199a-5p expression unveiled a pattern of induction in response to inflammation and apoptosis in BSS of UA. The expression pattern of miR-146b-5p and miR-199a-5p in BSS of UA compared with healthy control were more like consequences than causes. Such a pattern was partly supported by the studies about the role of miR-146b in inflammation. There were evidences that pro-inflammatory cytokines could induce the upregulation of miR-146b which in return extenuate inflammation as part of a negative feedback regulation loop [106]. In addition, the miR-146b regulatory circuit just fine-tuned inflammation related signaling, rather than totally abrogating the signal [106]. Whether apoptosis induced the upregulation of miR-199a-5p and miR-199a-5p functioned as a negative feedback regulation loop to inhibit apoptosis was worth further exploring. 

The present study revealed that microRNAs and mRNAs of PBMCs might be used as biomarkers for BSS of UA patients. Compared with healthy controls, levels of miR-146b-5p and miR-199a-5p were significantly higher, while levels of CALR and TP53 were significantly lower in BSS of UA patients. These miRNAs and mRNAs were closely related with BSS of UA patients. They might serve as biomarkers for distinguishing BSS of UA patients from healthy controls. To confirm this result, miR-146b-5p and miR-199a-5p and their downregulated target genes (CALR and TP53) were selected to validate the expression level in an independent cohort of 30 BSS of UA patients, 30 non-BSS of UA patients and 15 healthy controls by qRT-PCR. Compared with healthy controls and non-BSS of UA patients, significant upregulation of miR-146b-5p and miR-199a-5p and downregulation of CALR and TP53 were proven. It confirmed the robustness of the expression pattern of miR-146b-5p, miR-199a-5p, CALR and TP53 in BSS of UA patients.

However, research limitations existed in our study. First, the sample size was small, and clinical studies with larger cohorts of BSS of UA patients and healthy controls were definitely required to extensively evaluate the miRNAs and mRNAs as practical biomarkers in comparison with the diagnostic criteria and scale of BSS, as well as the false-positive rate. Second, there was lack of validation by Western blot. Because TCM syndrome differentiation was based on a collection of multiple symptoms and signs, the related biomedical mechanisms were likely quite complex. Thus, various miRNAs, genes, and intricate interactions were contained in the results, which made short time validation of results difficult. Nonetheless, the present study laid the groundwork for future efforts to identify and develop miR-146b-5p, miR-199a-5p, CALR and TP53 as a novel class of blood-based biomarkers for BSS of UA patients. According to these results, larger cohort investigations were designed to validate the diagnostic sensitivity and specificity of miR-146b-5p and miR-199a-5p as biomarkers. In addition, future studies will be performed to clarify the pathophysiological role of circulating miRNAs during pathogenesis of BSS of UA patients. 

## 5. Conclusions

In general, the present study revealed that miR-146b-5p, miR-199a-5p, CALR, and TP53, which were related to the negative feedback regulation loop to attenuate inflammation and apoptosis, were significant biomarkers of BSS of UA patients. The systems biology-based miRNA and mRNA expression biomarkers for BSS of UA may be useful for further stratification of UA patients when making decisions on treatments.

## Figures and Tables

**Table 1:** The grading system in quantifying blood stasis syndrome diagnosis standards.

Signs and symptom	Point
Purple tongue	(less severe) 8, (more severe) 10
Resistance to pressure in lower abdomen	(less severe) 8, (more severe) 10
Choppy pulse	10
Dark stool (Melena)	10
Pathogenic nodules	10
Distended veins under tongue	(less severe) 8, (more severe) 10
Irregular pulse	8
No pulse	10
Distended veins in abdominal wall	10
Hypodermal ecchymoses	(less severe) 8, (more severe) 10
Dark menstrual blood with clots	(less severe) 8, (more severe) 10
Persistent angina pectoris	10
General fixed pain	8
Dark red lips and gums	6
Small vessels	5
Numb extremities	5
Surgery history	5
Mucosal membrane of palate (+)	(less severe) 4, (more severe) 5
Paralysis in extremities	(less severe) 5, (more severe) 7
Psychiatric abnormality	(Irritability) 4, (Mania) 8
Rough skin	(less severe) 4, (more severe) 5
Complete blood viscosity (+)	10
Blood plasma viscosity (+)	5
External clot net weight (+)	10
External clot total weight (+)	8
Increase in platelet aggregation	10
Abnormality in blood clot elasticity	8
Microcirculation obstruction	10
Hemodynamics obstruction	10
Decrease in fiber dissolution activity	10
Resistance in platelet release	10
Pathogenic scan (+) for blood stasis	10
Blood vessel obstruction by new technology analysis	10

Grades <19 points are categorized as non-BSS. Grades 20–49 points are categorized as less severe BSS. Grades >50 points are categorized as more severe BSS [16, 22].

**Table 2:** The forward primers of miRNAs for amplification.

Accession	Name	Sequence	
MIMAT0002809	hsa-miR-146b-5p	Forward	5′-TGAGAACTGAATTCCATAGGCT-3′
MIMAT0000231	hsa-miR-199a-5p	Forward	5′-CCCAGTGTTCAGACTACCTGTTC-3′
X07425.1	Human U6 snRNA	Forward	5′-CGCAAGGATGACACGCAAATTC-3′

**Table 3:** The primers of mRNAs for amplification.

Accession	Name	Sequence	
NM_004343.3	CALR	Forward	5′-GGCTATGTGAAGCTGTTTCCTAAT-3′
		Reverse	5′-GTTCTTGCCCTTGTAGTTGAAGAT-3′
BC003596.1	TP53	Forward	5′-GGAGTAGGACATACCAGCTTAGATTT-3′
		Reverse	5′-TACCTAGAATGTGGCTGATTGTAAAC-3′
NG_007073.2	GAPDH	Forward	5′-CAAAGTTGTCATGGATGACC-3′
		Reverse	5′-CCATGGAGAAGGCTGGG-3′

**Table 4:** Clinical characteristics of subjects of microarray analysis.

	UA with BSS (*n* = 5)	Controls (*n* = 5)	*P* value
Male gender (*n*)	3 (60%)	3 (60%)	
Age (years)	61.60 ± 4.93	61.00 ± 4.36	0.596
BMI (kg/m^2^)	23.18 ± 3.89	23.24 ± 1.62	0.975
Active smoker (*n*)	1 (20%)	1 (20%)	
BSS grades	72.80 ± 10.47	0	
Number of vessels (*n*)	3.40 ± 0.89	0	
Hypertension (*n*)	4 (80%)	0	0.048
Type 2 diabetes mellitus (*n*)	0	0	
Total cholesterol (mmol/L)	3.96 ± 1.45	4.02 ± 0.79	0.347
LDL cholesterol (mmol/L)	2.33 ± 1.01	2.51 ± 0.60	0.743
HDL cholesterol (mmol/L)	1.33 ± 0.35	1.30 ± 0.30	0.874
Triglycerides (mmol/L)	1.15 ± 0.50	1.09 ± 0.34	0.815
CRP (mg/L)	3.64 ± 3.25	0.94 ± 0.09	0.018
Concurrent medication (*n*)			
Antiplatelet	100%	0	0.008
Beta-blocker	4 (80%)	0	0.048
ACEI/ARB	4 (80%)	0	0.048
Calcium-channel blockers	3 (60%)	0	0.167
Nitrates	4 (80%)	0	0.048
Statin	4 (80%)	0	0.048

Data represents means ± SEM. Abbreviations: ACEI: angiotensin-converting enzyme inhibitor; ARB: angiotensin receptor blocker; BMI: body mass index; BSS: blood stasis syndrome; HDL: high-density protein; CRP: C-reactive protein; LDL: low-density protein.

**Table 5:** Clinical characteristics of subjects of qRT-PCR.

	UA with BSS (*n* = 30)	UA with non-BSS (*n* = 30)	Controls (*n* = 15)	*P* value
Male gender (*n*)	15 (50%)	15 (50%)	8 (53.3%)	0.974
Age (years)	57.06 ± 5.62	57.67 ± 6.43	56.87 ± 6.70	0.896
BMI (kg/m^2^)	24.58 ± 1.52	24.10 ± 2.13	23.63 ± 1.52	0.231
Active smoker (*n*)	17 (56.7%)	18 (60%)	6 (40%)	0.429
BSS Grades	73.93 ± 2.91	14.03 ± 2.22	0	
Number of vessels (*n*)	1.53 ± 0.94	1.57 ± 0.95	0	
Hypertension (*n*)	22 (73.3%)	23 (76.7%)	0	0.000
Type 2 diabetes mellitus (*n*)	14 (46.7%)	15 (50%)	0	0.001
Total cholesterol (mmol/L)	4.19 ± 0.83	4.19 ± 0.95	3.71 ± 0.76	0.172
LDL cholesterol (mmol/L)	2.60 ± 0.59	2.55 ± 0.58	2.37 ± 0.54	0.436
HDL cholesterol (mmol/L)	1.13 ± 0.21	1.12 ± 0.21	1.31 ± 0.29	0.032
Triglycerides (mmol/L)	1.79 ± 0.89	1.89 ± 1.04	1.03 ± 0.27	0.001
CRP (mg/L)	3.93 ± 2.21	3.98 ± 2.26	2.73 ± 2.05	0.122
Concurrent medication (*n*)				
Antiplatelet	100%	28 (93.3%)	0	0.000
Beta-blocker	20 (66.7%)	20 (66.7%)	0	0.000
ACEI/ARB	18 (60%)	16 (53.3%)	0	0.000
Calcium-channel blockers	13 (43.3%)	15 (50%)	0	0.001
Nitrates	22 (73.3%)	20 (66.7%)	0	0.000
Statin	26 (86.7%)	24 (80%)	0	0.000

Data represents means ± SEM. Abbreviations: ACEI: angiotensin-converting enzyme inhibitor; ARB: angiotensin receptor blocker; BMI: body mass index; BSS: blood stasis syndrome; HDL: high-density protein; CRP: C-reactive protein; LDL: low-density protein.

**Table 6:** Differentially expressed miRNAs in UA patients with BSS versus healthy controls.

ID	Name	UA with BSS/control ratio	*P* value
PH_mr_0001929	hsa-miR-221-3p	1.58	0.0440
PH_mr_0000996	hsa-miR-130a-3p	1.55	0.0091
PH_mr_0001948	hsa-miR-199a-5p	1.41	0.0438
PH_mr_0003258	hsa-miR-223-3p	1.38	0.0324
PH_mr_0002734	hsa-miR-199a-3p	1.36	0.0171
PH_mr_0000023	hsa-miR-126-3p	1.27	0.0443
PH_mr_0004749	hsa-miR-4485	1.24	0.0443
PH_mr_0000176	hsa-miR-146a-5p	1.18	0.0328
PH_mr_0000566	hsa-miR-151a-5p	1.17	0.0387
PH_mr_0000751	hsa-miR-146b-5p	1.15	0.0227
PH_mr_0000446	hsa-miR-22-3p	1.15	0.0421
PH_mr_0001186	hsa-miR-150-5p	0.95	0.0165
PH_mr_0008005	hsa-miR-1307-5p	0.94	0.0051
PH_mr_0001511	hsa-miR-151a-3p	0.94	0.0147
PH_mr_0000436	hsa-miR-92a-3p	0.94	0.0301
PH_mr_0001750	hsa-miR-589-5p	0.94	0.0188
PH_mr_0001629	hsa-miR-484	0.88	0.0406
PH_mr_0001844	hsa-miR-326	0.87	0.0068
PH_mr_0001893	hsa-miR-30d-5p	0.86	0.0228
PH_mr_0000911	hsa-miR-129-1-3p	0.86	0.0379
PH_mr_0001753	hsa-miR-196a-3p	0.85	0.0159
PH_mr_0003163	hsa-miR-423-3p	0.83	0.0203
PH_mr_0002404	hsa-miR-625-5p	0.80	0.0360
PH_mr_0004525	hsa-miR-4459	−0.88	0.0225
PH_mr_0004803	hsa-miR-4667-5p	−1.02	0.0419

**Table 7:** The pathways related to the upregulated mRNAs between UA patients with BSS and healthy controls.

KEGG pathway	Number of genes	*P* value
hsa04621: NOD-like receptor signaling pathway	9	0.0024
hsa04210: apoptosis	9	0.0182
hsa03040: spliceosome	11	0.0228
hsa04640: hematopoietic cell lineage	8	0.0469
hsa05130: pathogenic Escherichia coli infection	6	0.0672
hsa03018: RNA degradation	6	0.0672
hsa04060: cytokine-cytokine receptor interaction	16	0.0790

**Table 8:** The pathways related to the downregulated mRNAs between UA patients with BSS and healthy controls.

KEGG pathway	Number of genes	*P* value
hsa05332: graft-versus-host disease	5	0.0121
hsa04612: antigen processing and presentation	7	0.0121
hsa04115: p53 signaling pathway	5	0.0722
hsa00280: valine, leucine, and isoleucine degradation	4	0.0812
hsa00510: N-Glycan biosynthesis	4	0.0901
hsa00532: chondroitin sulfate biosynthesis	3	0.0913

**Table 9:** The degrees of hubs of the PPI network.

Genes	Degree	Genes	Degree
MAPK14	20	E2F1	14
AKT1	18	SMAD3	14
EP300	17	GNB1	14
HDAC1	17	MYC	14
TP53	16	SRC	14
